# In-house three-dimensional printing for surgical planning: learning curve from a case series of temporomandibular joint and related disorders

**DOI:** 10.3389/fvets.2024.1347107

**Published:** 2024-02-06

**Authors:** Miguel R. Godinho, Lisa A. Mestrinho

**Affiliations:** ^1^Faculty of Veterinary Medicine, University of Lisbon, Lisbon, Portugal; ^2^Centre for Interdisciplinary Research in Animal Health (CIISA), Faculty of Veterinary Medicine, University of Lisbon, Lisbon, Portugal; ^3^Laboratório Associado para Ciência Animal e Veterinária (AL4AnimalS), Lisbon, Portugal

**Keywords:** 3D printing, temporomandibular joint ankylosis, stereolithography, surgical cutting guide, workflow, oromaxillofacial surgery, open-source software, computer-aided surgical planning

## Abstract

Three-dimensional (3D) printed models can improve the understanding of the structural anatomic changes in cases of temporomandibular joint ankylosis and pseudoankylosis leading to closed jaw locking. Their use in pre-surgical planning and intraoperative guidance has been reported, contributing to the predictability and success of these surgery procedures, which can be quite complex, especially in small animal patients. The use and production of 3D tools and models remain challenging and are so far limited to institutions with high (economical and human) resources. This study aims to propose simplified workflows using open-source software to facilitate an in-house 3D printing process. To illustrate this, three cases of temporomandibular joint ankylosis and one of pseudoankylosis were reviewed, where in-house 3D printed models were used for client communication and surgical management. The 3D models were segmented from computed tomography and printed via stereolithography. They were used to support discussion with clients (*n* = 4), to allow surgeons to pre-surgical plan and practice (*n* = 4) and for intraoperative guidance during surgery (*n* = 2). Surgical cutting guides were produced in one case to improve precision and define more accurately osteotomy lines. It is essential to consider the initial time and financial investment required for establishing an in-house 3D printing production, particularly when there is a need to produce biocompatible tools, such as surgical cutting guides. However, efficient and streamlined workflows encourage the integration of this technology, by accelerating the printing process and reducing the steep learning curves, while open-source software enhances accessibility to these resources.

## 1 Introduction

Three-dimensional (3D) printing has been gaining increasing relevance in the medicinal field (1). In veterinary medicine, it is currently used for various purposes, including models for teaching and surgical training, patient-specific devices such as cutting guides and implants. More recently, due to the complex and delicate anatomy of the head, this technology has become prominent in the specialized field of dentistry and oromaxillofacial surgery. Its application aims to improve patient outcomes, reduce surgical times, and minimize complications (2).

Temporomandibular joint (TMJ) ankylosis results from the formation of osseous or fibrous connective tissue within the articulation (true ankylosis) or in the surrounding structures (false ankylosis or pseudoankylosis) (3), leading to closed jaw locking (4).

Advanced diagnostic imaging is essential for the diagnosis and characterization of oromaxillofacial diseases, especially TMJ ankylosis and serves a crucial purpose in preoperative planning (5). Various methods can be used, primarily computed tomography (CT) and cone-beam computed tomography, but also magnetic resonance imaging (6). However, these imaging modalities, even with 3D reconstruction, remain limited when it comes to displaying spatial relationships for complex anatomical features, lacking a tactile manipulation component that could help mitigate these difficulties. To overcome these limitations, 3D printing technology offers a solution by translating conventional imaging into a 3D printed model (7).

There are 3D printers of all sizes, shapes, and types, as well as a variety of printing techniques (8). The materials used range from plastics to resin, sand, ceramics, metal, or a mixture of these, even including organic materials. They can be used either in liquid, solid, or powder form, depending on the printing technique employed (9). Stereolithography (SLA) is the oldest 3D printing technology (10). It uses lasers to polymerize resin held in a liquid state, to make the design 3D structure (11). This modality advantages include high speed, strong printed structure, very high levels of resolution and accuracy, and stepped layer free texture (9, 12). As a result of this technology advancements, SLA technique is now available in small, relatively low-cost printers, which has made 3D printing more accessible and affordable (7).

3D printed models can allow surgeons, students, and clients to improve their understanding of the anatomy and diseases of the oral and maxillofacial complex through tactile and visuospatial perception (13, 14). This approach has been described to be beneficial in the surgical planning of the TMJ (3, 4, 6, 7, 13–16).

Design and production of 3D tools involve inherent complexity, given the multifaceted nature of the process. A comprehensive understanding of various aspects is required, ranging from mathematical principles to intricate graphical representations. The primary objective of this study is to describe and present streamlined workflows to facilitate in-house creation of 3D models and simple surgical cutting guides, utilizing open-source software. The advantages and limitations on using this technology for surgical planning of the TMJ are also reviewed.

## 2 Methods

The study included TMJ ankylosis and pseudoankylosis cases presented at the University of Lisbon’s Veterinary teaching hospital between 2021 and 2023. Inclusion criteria encompassed a head CT scan and the use of 3D printing for preoperative study and planning. Cases where 3D printed models were absent from the surgical planning were excluded.

### 2.1 Image acquisition

Pre-operative images were acquired using a Toshiba Astelion TSX-034A 16 CT scanner under general anesthesia, with 110 kV, and 200 mAs, in a bone window and a soft tissue window, a slice thickness ranging from 0.5 to 0.6 mm and a pitch of 0.9. Image acquisition was performed in Digital Imaging and Communications in Medicine (DICOM) image format and stored as .dcm files for analysis using the open-source software Horos (Horos Project, Annapolis, MD, United States).

### 2.2 3D volume rendering and segmentation

**Figure 1:**
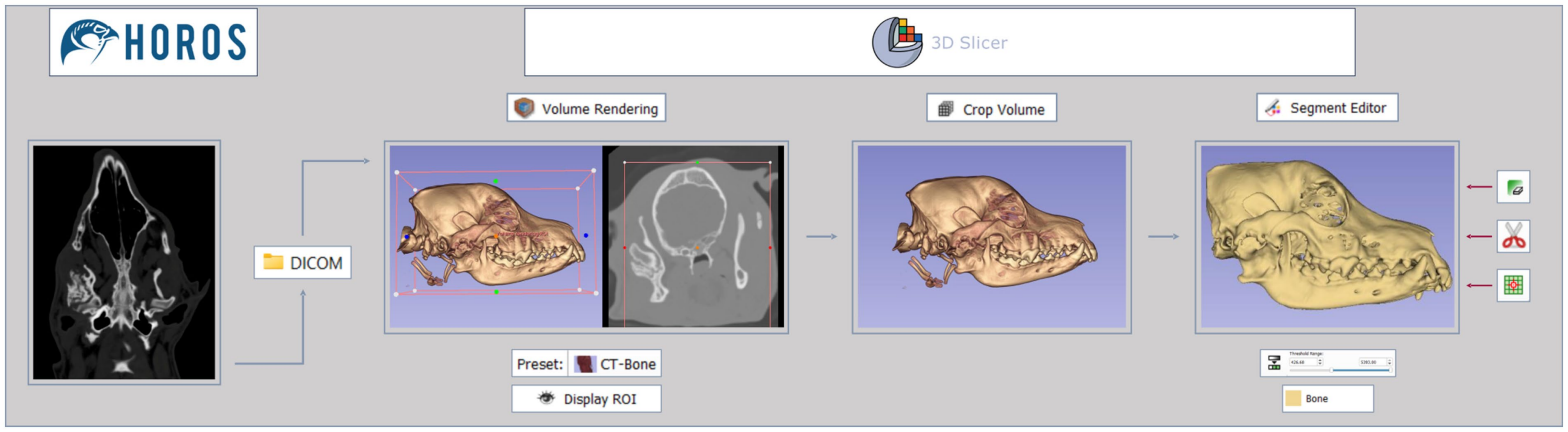
Segmentation process workflow. Images in DICOM file format, interpreted via Horos software, are imported to 3D slicer software. The data then goes through 3 different modules—volume rendering, crop volume, and segment editor—to create a final 3D virtual model. Tools used are shown under or on the side of the images.

The DICOM data was then converted into 3D models using a CAD open-source software—3D slicer,^1^ following the workflow diagram illustrated in Figure 1. After importing the data, by drag and drop, the 2D CT scan images were converted into a 3D image, using the rendering volume module. For this, a CT-bone preset was selected, which has a specific threshold and color to help visualize the intended aspects of the data. Still in this module, the segmentation process began, through the determination of the region of interest (ROI). A rectangular prism volume was defined to encompass all relevant structures for segmentation. In this study, emphasis was placed on the examination of the bony structure of the skull, with particular focus in the TMJ region. The “Display ROI” tool was activated, and limits were manually determined across both CT and 3D tabs until the ideal ROI was achieved. The crop volume module was then used to finalize the determined ROI, selectively framing only the desired structures. The software’s default settings were maintained, and the changes were confirmed by clicking the “Apply” button. After this, the “Display ROI” tool was deactivated. This initial step is important since the maximum size of the object must fit the maximum size of the printer. To maintain the real dimensions of the structures in the 3D printed model, in cases 2 and 3 the most caudal part was not included in the segment, as the original size of the cranium would exceed the dimensions allowed by the printer used.

The segmentation process was completed using the segment editor module, where threshold range for bone was selected using the threshold tool. During this stage, the emphasis shifted from spatial considerations to highlighting the intensity of the structures, particularly focusing on the bone. To perform this a new empty segment was added, and the bone range in Hounsfield units was established between 500 and 3,000, as previously described in the literature (17). The lower threshold values were then fine-tuned through visual assessment. This adjustment process varied in each case, owing to differences in factors such as size, skeletal structure, and composition among individuals. To accomplish this, the bone within the ROI was carefully evaluated in the cross-sectional images—transverse, sagittal, and dorsal—allowing for slight modifications to the lower threshold values. Visual judgment is the most commonly and easily employed criterion for bone segmentation, particularly in human models (18, 19). When working with high-resolution images, the visual-based method produces a more reasonably accurate 3D reconstruction, outperforming other automated methods to a certain extent (20). The lower threshold values ranged from 426.68 to 602.97 across cases. The higher threshold values were set to their maximum, given that the ROI was targeting the structures with the highest radiological density (bone). The generated model was displayed by selecting the “Show 3D” button.

In most cases there was also a need to remove non-relevant structures, such as non-cranial bones and other objects with similar radiological density (e.g., endotracheal tube, nasogastric tube, etc.), from the model. Following tools were used: scissors, islands, and erase. The scissors tool was utilized to cut through the entire segment from the current viewpoint, erasing everything inside the outline. This facilitated the elimination of larger non-relevant structures. Default settings, including operation, shape and slice cut, were maintained. The islands tool was employed to eliminate structures not connected to the main segment. The “remove islands” option was applied, eliminating segments smaller than a specified minimum size—4,000 voxels. The erase tool played a crucial role in addressing small aspects that the scissors tool could not effectively handle, through the precise exclusion of data slice by slice. Additionally, it proved essential in assisting the functionality of the islands tool by isolating relevant structures from those considered non-relevant. These three tools were used to complement each other and not in a specific order. After the segmentation process, the designated 3D models were saved in STL (stereolithography) file format to be uploaded to the printing software.

### 2.3 Surgical cutting guides

**Figure 2:**
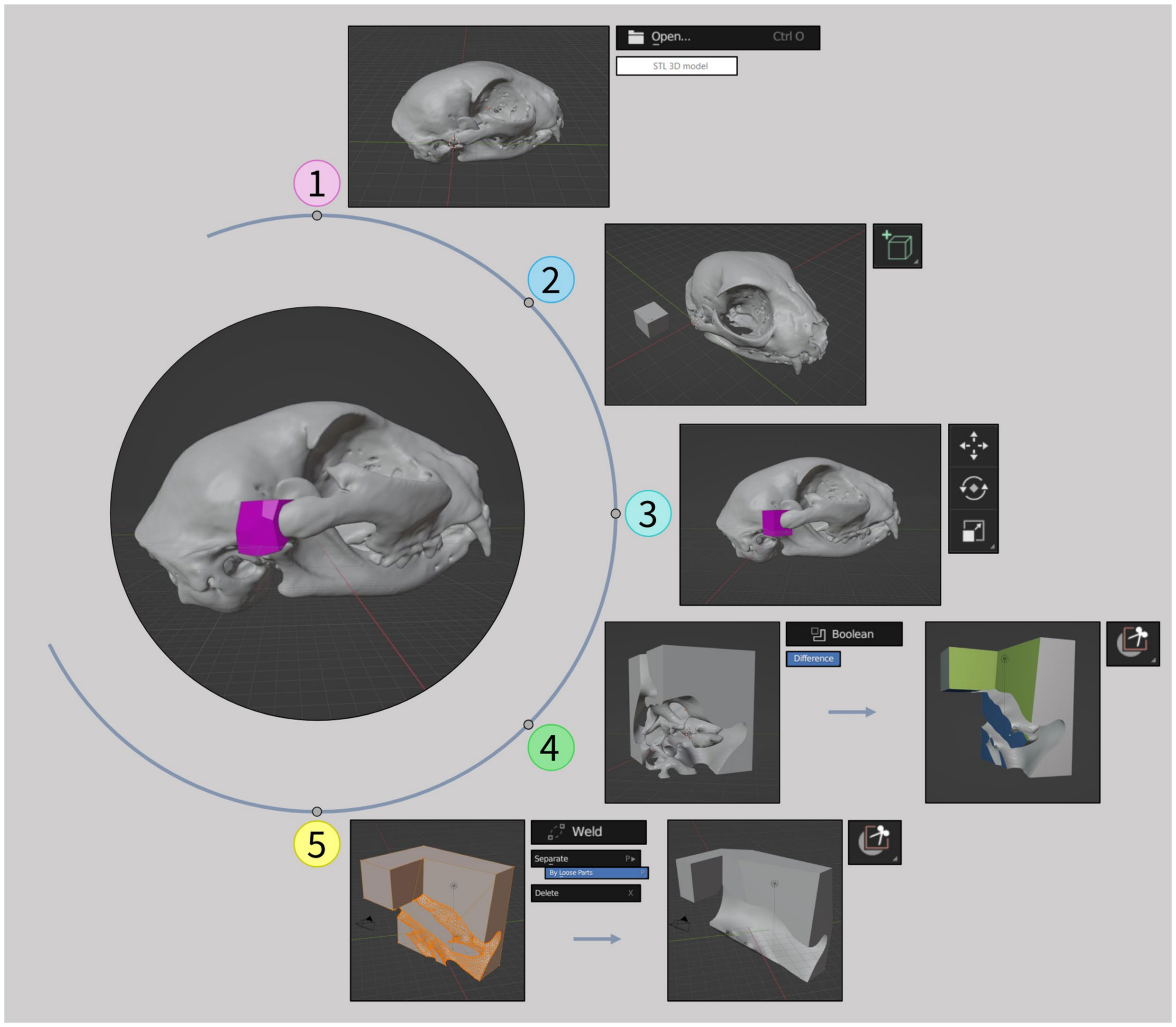
Surgical cutting guides development workflow. (1) Importation of the 3D model in STL file format (previously created in 3D slicer) to Blender software. (2) Addition of a cube to the mesh. (3) Positioning of the cube at the injury site. (4) Creation of a socket-like void and isolation of the primary segment. (5) Creation of solid segments, elimination of unrelated parts and final refinements of the guide. Tools used are shown on the side of the images.

The surgical cutting guides were meticulously crafted using the open-source 3D computer graphics software Blender version 3.6.5 LTS (Blender Foundation). These guides were customized to match the patient’s anatomy with precise dimensions for the intended surgical cuts. To achieve this, the 3D model (created previously in 3D slicer) was first imported into Blender (Figure 2, step 1). Then, in object mode, a cube was added to the mesh (Figure 2, step 2) and precisely positioned at the injury site (Figure 2, step 3). A subtraction operation was performed using the Boolean tool to remove the cube’s points that overlapped with the injury, creating a socket-like void (Figure 2, step 4). Switching to sculpt mode, the box trim tool was used to isolate the primary segment (the guide), without compromising its intended shape. Returning to object mode, the weld tool was employed to merge closely located vertices, ensuring the creation of solid segments. Any remaining parts unrelated to the guide were eliminated using the “separate by loose parts” and “delete” commands in the edit mode, resulting in the final guide segment (Figure 2, step 5). Further refinements of the external dimensions were made using the box trim tool once again, enhancing both its aesthetics and functionality. This adjustment made it easier to grip, addressing the issue of its diminutive size. This process was repeated for each guide. Final files were exported in STL file format for uploading to the 3D printing software.

### 2.4 3D printing

**Figure 3:**
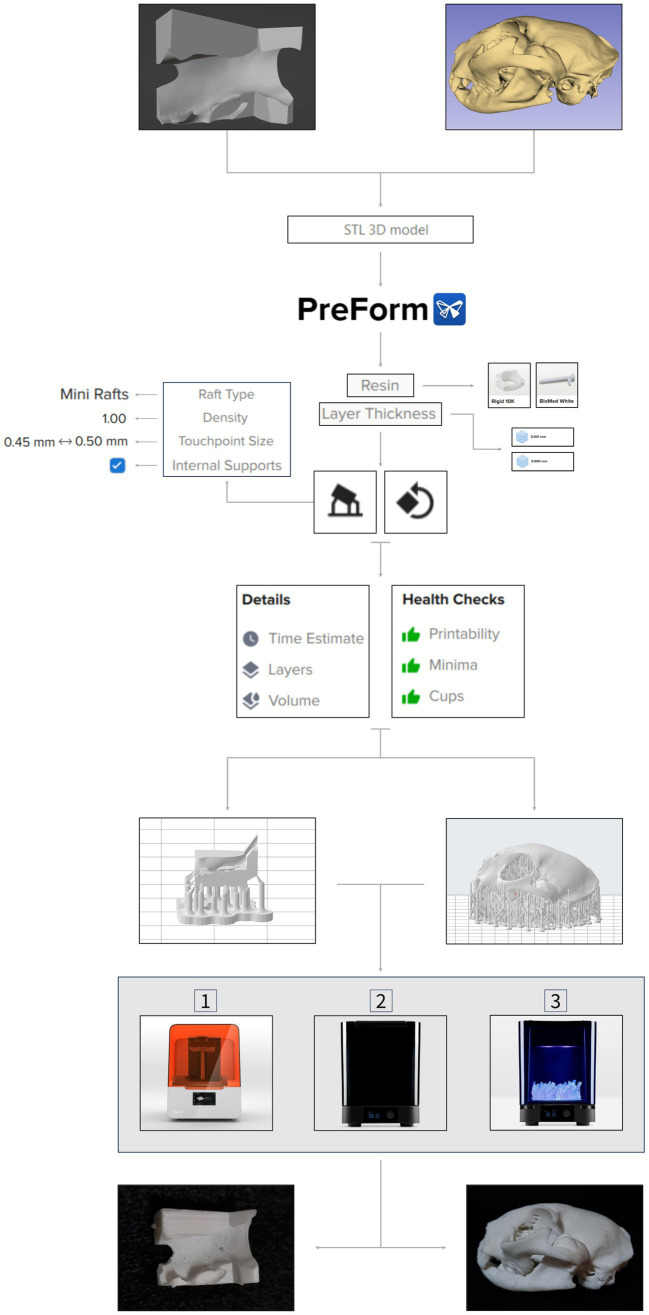
3D printing process workflow. Both 3D anatomical models and surgical cutting guides in STL file format are imported to PreForm software. Resin type and layer thickness are selected, followed by orientation and support structure design processes. Once health checks are done, the 3D virtual pieces are 3D printed by an SLA printer—Formlabs 3B (1). Next, the pieces go through a washing machine (2), for 20 min, and a cure machine (3), for 60 min. After this post-processing, final products are obtained, and they can now be used for pre-surgical planning or be sterilized to be part of the surgery. Tools’ features are shown on the side of the workflow. Partial images courtesy and copyright of Formlabs Inc.

An SLA printer (Form 3B, Formlabs, United States) was used to print the 3D tools. A rigid white resin (Rigid 10K) was used for the models, while biocompatible resin (Biomed White) was used for the guides. The previous obtained STL files were uploaded to the 3D printer software PreForm (Formlabs, United States) which automatically converted them to the printer’s directions. Following steps included choice of the resin, optimization of the model’s orientation, using the orientation tool, to minimize both the material required and the printing time, and design of the support structures, using the supports tool, to ensure stability during printing. While the software integrates automated processes for these features, their performance may not always meet optimal standards. In this study, the design of the support structures was automated, based on predefined settings (raft type, density, touchpoint size, internal supports); however, orientation was carried out manually. This task involved translation and rotation of the models until error-free printability was ensured. Consideration was also given to post-processing, specifically addressing the removal of support structures after printing. In the support structure configurations, mini rafts were chosen with a density of 1.00. Mini rafts had a touchpoint size of 0.50 mm in the small models and a touchpoint size of 0.45 mm in the larger models. Internal supports were also added. The smaller models were printed with a 0.050 mm layer thickness, while the larger ones utilized a 0.100 mm layer thickness. Before printing, automatic health checks were carried out to assess factors such as the number of the model’s cups and minima, to evaluate the printability. The printing process for the surgical cutting guides was identical as the one used for the small models, with the purpose of intraoperative use on the actual lesion after sterilization. Printing time, number of layers, and resin volume consumed were recorded for each 3D piece. The 3D printing process workflow used in this study is illustrated in Figure 3.

After printing, support structures were manually removed using pliers or by hand, followed by minimal post-processing. This involved a 20 min immersion in propyl alcohol (solvent) and a subsequent 60 min cure at 60°C under UV light, utilizing the same printer supplier (Formlabs) washing and cure machines (see Figure 3). Some inner supports proved challenging to eliminate entirely. Cleaning of printing equipment—the build platform and steel print surface—was carried out using the same type of alcohol and a steel spatula.

## 3 Results

Three cases of TMJ ankylosis and one case of pseudoankylosis were included in this study for which 3D printed models were produced. In all four, the models were used to discuss the surgical strategy with the clients during consultation and to plan the surgical procedure. In two cases models were used intraoperatively and in one case both model and surgical guides were designed and produced for intraoperative guidance.

### 3.1 Case presentation

Case 1: an adult female domestic shorthair intact cat was presented for consultation with an inability to open the oral cavity, complete absence of solid food prehension, and severe dysphagia for liquids. The animal was in an advanced state of malnutrition and dehydration, weighing 1.9 kg, without prior medical history. After initial approach and improvement of the clinical condition, CT scan revealed left TMJ ankylosis, with fusion between the coronoid process, the caudal portion of the zygomatic arch and the zygomatic process of the temporal bone.

Case 2: a 9 months-old female mixed-breed intact dog was referred for consultation with a diagnosis of right TMJ ankylosis based on radiography. History included incapacity to open the oral cavity. No previous treatments were performed. CT scan indicated both ankylosis and pseudoankylosis, with synostosis between the zygomatic process of the temporal bone, the temporal process of the zygomatic bone, the masseteric fossa, and the mandibular condylar process, on the right side. Additionally, malocclusion was noted: right mandibular distoclusion, horizontal maxillomandibular asymmetry.

Case 3: a 1 year-old female Cane Corso intact dog was referred for consultation with inability to open the oral cavity and asymmetric face (left side slightly deformed). The dog had a history of a bite injury to the neck and head at a young age. CT scan showed pseudarthrosis and pseudoankylosis of the left TMJ, with possible secondary fibrosis, resulting from an irregular exostosis present laterally with small fragments.

Case 4: an 8 months-old female European Shorthair intact cat was referred for consultation with a history of being unable to open its oral cavity. It had previously received anti-inflammatory and antibiotic treatments, which were unsuccessful. Examination revealed low body condition score and complete inability to open its mouth. CT scan demonstrated bilateral and symmetric temporomandibular ankylosis, possibly to old mandibular condylar head and temporal bone fractures with joint space collapse.

### 3.2 3D printing process

The 3D printing of all models and surgical cutting guides was successful. Specific printing parameters for the 3D pieces, including printing time, number of layers and volume of resin consumed, are as follows:

- Case 1: 9 h and 40 min to print, 1,154 layers and 50.61 mL of resin consumed.
- Case 2: 13 h and 40 min to print, 1,536 layers and 164.75 mL of resin consumed.
- Case 3: 20 h and 12 min to print, 1,829 layers and 291.94 mL of resin consumed.
- Case 4: 8 h and 11 min to print, 1,069 layers and 43.22 mL of resin consumed.
- Right surgical cutting guide: 1 h and 09 min to print, 272 layers and 0.70 mL of resin consumed.
- Left surgical cutting guide: 1 h and 15 min to print, 294 layers and 0.78 mL of resin consumed.

Labor time estimates, encompassing both learning and acquisition results, were recorded for each software and 3D printing process. The overall learning time to gain basic proficiency with all software was approximately 1 week. Image study using Horos took approximately 2 h and 30 min per case, 3D rendering and segmentation using 3D slicer took approximately 2 h per case, surgical cutting guide design took approximately 4 h, and printing preparation took between 15 to 30 min. Knowledge about software operation was acquired through online tutorials.

### 3.3 Surgical planning and surgical cutting guides

In cases 1 and 2, the models were used preoperatively to plan the osteotomy lines. The same models were then sterilized with the osteotomy lines marked and taken to the surgical setting to assist in the identification of the pre-planned osteotomy lines.

**Figure 4:**
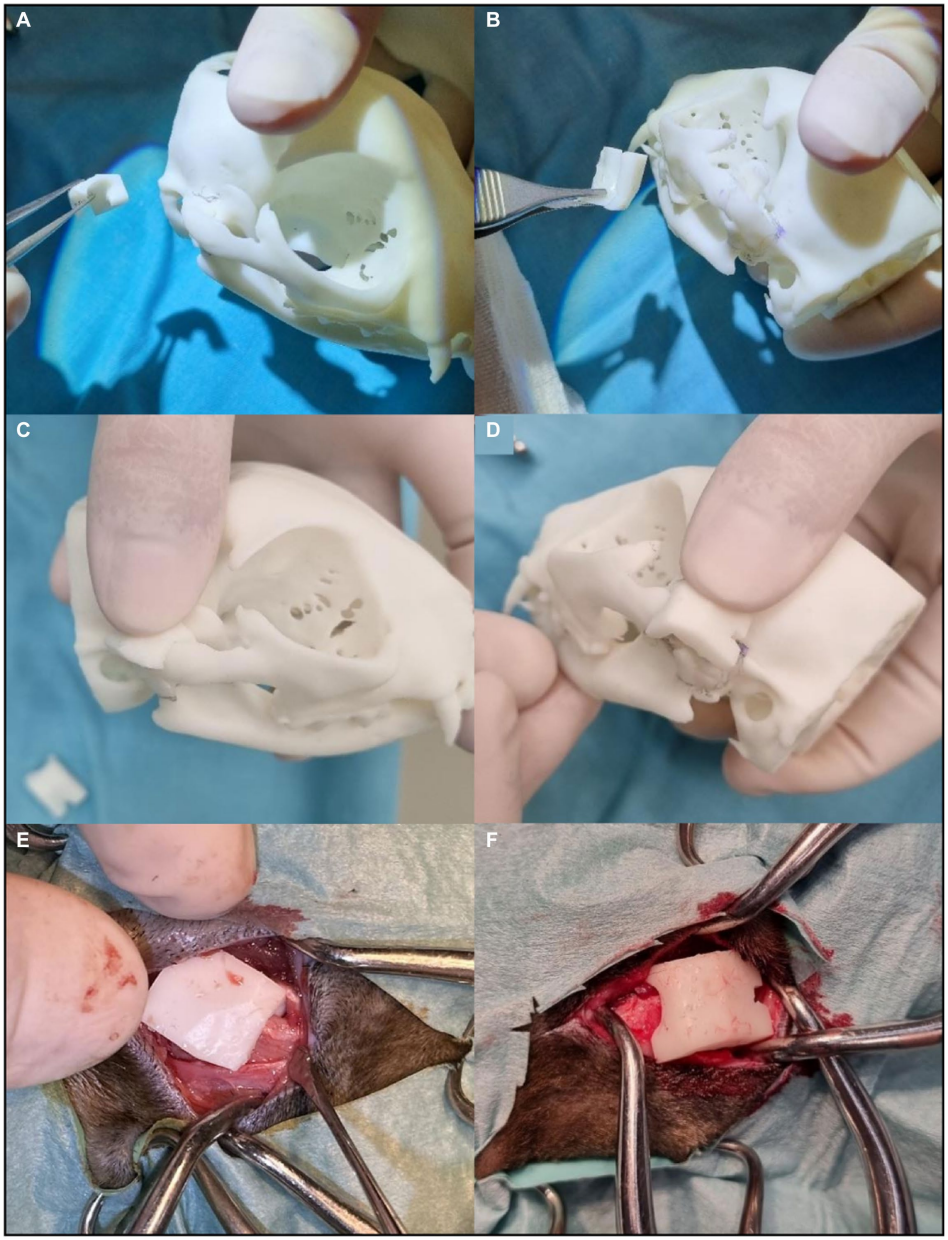
Surgical cutting guides application in surgery. **(A)** Right TMJ surgical cutting guide. **(B)** Left TMJ surgical cutting guide. **(C)** The right guide fits perfectly on the model. **(D)** The left guide fits perfectly on the model. **(E)** Introduction of the right guide at the intervention site. **(F)** Introduction of the left guide at the intervention site. Both surgical cutting guides fit naturally on the real structures of the animal. The osteotomy blade was aligned to the plane defined by the edges of the guide.

In case 4, surgical cutting guides were created for a more precise and careful removal of both ankylosis segments. These guides were attached to the segments selected to be excised, using a small bone fragment reduction forceps. The edges of the guides determined the orientation and alignment of the osteotomy blade. The production process for these guides complied with the workflow described in the methods section, as depicted in Figure 2. The intraoperative application is illustrated in Figure 4.

### 3.4 Clinical decisions and communication

When communicating with the clients, the models were also used to visually represent and explain the existing disease, as well as to alert about potential complications and expected outcomes. Among the cases featured in this study, all but one chose to proceed with surgical treatment. The reason for the exception (case 3) remains unknown.

Before each surgical procedure, the surgeon studied the corresponding 3D anatomical model to enhance a more precise intervention and reduce the risk of complications. During surgery, the models, in addition to providing anatomical references, served as educational tools for students and doctors, aiming to enhance their understanding of oromaxillofacial anatomy, diseases, and surgical approaches.

### 3.5 Investment

Printing the models incurred costs ranging from 15.90€ to 107.37€, while the surgical cutting guides had a total cost of 0.49€. In addition to these expenses, there was an initial investment of approximately 8,500€ for essential equipment, which included the 3D printer, resin tanks, and washing and curing machines. Different types of resin require different resin tanks, which are considered consumables, just like the resin itself. Typically priced at around 160€, these tanks are specifically used for each resin and are non-interchangeable. The cost of the computer was not included in these overall expenses.

In addition to operational expenses, it’s essential to consider costs associated with machine wear and tear, maintenance parts, and the eventual replacement of machinery due to aging. These factors contribute to the overall lifecycle expenses and should be carefully accounted for in financial planning and decision-making processes.

## 4 Discussion

This case series illustrates the potential applications, advantages, and limitations of 3D printing technology in the diagnostic and therapeutic approach to TMJ ankylosis and pseudoankylosis and in veterinary oromaxillofacial surgery. Two dogs and two cats were included in the study. Clinical presentations and diagnosis features observed align with those documented in previously published literature (21, 22). Surgical approach was generally similar, but each case is different in terms of specific features of the lesions presented; thus, different strategies are usually needed. The 3D printed models were incorporated in both clinical communication and the surgical setting. These cases also illustrate the learning curve of this team in establishing an in-house production of individual models for surgical guidance and surgical cutting guides.

High-quality images are indispensable for both accurate diagnosing and the creating of precise, high-resolution 3D models. Achieving this level of detail requires the use of advanced imaging techniques. The algorithms chosen for image capture allowed a meticulous image analysis to facilitate the identification of the lesion’s features, location, and side. This level of detail is crucial for the pre-surgical planning. The finer the image slices used, the greater the precision and accuracy of the resulting 3D printed model.

While 3D printed models offer valuable insights, it is essential to underscore that the comprehensive characterization of the TMJ ankylosis cannot solely rely on these models. Advanced diagnostic imaging, notably through CT and CBCT, remains imperative. These imaging techniques ensure a thorough examination, capturing fine details that might be overlooked in 3D printed representations. Therefore, a synergistic approach that integrates both advanced imaging and 3D printing is crucial for a comprehensive understanding of TMJ conditions.

Individualized pre-surgical planning, through 3D anatomical models with patient-specific anatomy and pathology, enables medical professionals to conduct a more detailed analysis of structures to be intervened, including relevant spatial relationships. This allows the surgeon to be more rigorous in planning of the surgical steps by choosing, adapting, practicing, and perfecting the most appropriate surgical procedure, simultaneously increasing his confidence. If necessary, preliminary simulations can be performed. Additionally, these 3D models can be sterilized. There are many biocompatible commercial resins available. In the context of 3D model production, prioritizing cost-effectiveness, the authors believe that investing in biocompatible resins may be deemed unjustifiable due to their higher costs compared to standard resins. This is because, once polymerized, these resins are heat resistant and can seamlessly undergo the sterilization process without dimensional changes (23). While the higher price of biocompatible resins can be justified for the low volume needed to produce surgical cutting guides, it is crucial to consider consistent production of a significant number of guides or other biocompatible devices to effectively offset the initial investment in both time and money.

In this case series, all cases opted for surgical treatment, except for case 3, where no justification was provided. Typically, animals displaying such relevant and severe clinical signs choose to undergo surgery. However, due to the limited size of the case series, it was not feasible to accurately assess the true extent of the models’ influence on clinical decision-making when compared to cases without access to the models. Therefore, it is not possible to infer whether the decision in case 3 was influenced by the models or not. Nonetheless, in the other cases, this approach demonstrated to be valuable in enhancing understanding and facilitating communication with clients. The hands-on manipulation and tangible visualization offered by these models provided a deeper insight into surgical complexity, potential complications, and expected outcomes. This not only addresses challenges associated with clients’ interpretation of complex images from advanced diagnostic imaging but also promotes a more thoughtful decision-making process.

The surgical cutting guides maximized the precision and accuracy of the surgical procedure in case 4. When applied, they allow optimized results both aesthetically and clinically, as well as shorter surgical times (6). In this case, the use of surgical cutting guides may have contributed to a potentially shorter surgical time, but drawing conclusive findings from a single case is challenging, given the absence of comparisons with other cases in this series. Despite this limitation, the authors believe that using these guides led to a quicker execution of the osteotomy step compared to relying solely on the 3D model. Additionally, at the conclusion of the procedure, these guides facilitated a verification process to ensure alignment between the initially drawn cutting lines in the model and the actual osteotomy lines in the real case. Their precise definition of the osteotomy lines significantly enhanced the efficiency of the procedure, mitigating the risk of serious complications. These complications include failure to resolve the TMJ block and overly deep cuts that could result in undesired damage to adjacent structures, such as the risk of intracranial injury due to a temporal bone fracture. In the cases where surgical cutting guides were not produced, the osteotomy lines were planned and designed based on the 3D-printed cranium model as part of the pre-surgical planning process. It should be noted, however, that it is crucial to maintain anatomical accuracy in the image dataset for achieving an ideal surgical outcome. Failure to do so may result in inadequate surgical margins and incompatibility of the surgical guides (3). Mastering the development of surgical cutting guides demands extensive training and should be pursued only after attaining proficiency and comfort in anatomical model development. Nevertheless, surgical cutting guides prove to be highly beneficial, constituting a suitable option for upcoming procedures involving such contours.

This study proposed and outlined several sequential workflows for the software and equipment required in the different stages of 3D model and simple surgical cutting guide development. The goal is to facilitate the establishment of in-house production for these tools. The various workflows, seen in Figures 1–3, are straightforward and can be easily repeated when using the same or equivalent items. Efficient and streamlined workflows are essential for the successful integration of 3D printing technology in clinical and surgical settings beyond academic environments. The 3D printing stages encompassed by these workflows include 3D volume rendering, segmentation, design, and printing processes. The adoption of such workflows offers distinct advantages in healthcare, such as the expedited production of patient-specific 3D models and the reduction of the steep learning curves often associated with complex design software. As a result, these streamlined processes lead to more efficient production, ultimately promoting the broader adoption of 3D printing technology in these critical domains and enhancing patient care and medical innovation. In this study, open-source software was employed; however, it is necessary to acknowledge the extensive array of available alternatives, whether freely accessible or subscription-based.

The selection of software should align with the project’s unique and specific requirements. Some offer more user-friendly interfaces and tools, leading to less labor-intensive processes. Regarding methods for separating parts of a model, both open-source and non-open-source software provide automatic workflows with varying levels of accuracy, in addition to manual options. In the open-source software used in this study, tools such as “mask segmentation” and “grow from seeds” enable the automatic and more accurate separation of different parts of a model, allowing for the selection and erasure of specific areas. However, these processes may take more time and were not applied in the present study. The authors believe that the tools used for deleting the necessary parts in this study did not compromise the accuracy of the final model and can be applied efficiently without sacrificing quality.

It is important to highlight that the skills developed in the course of this research study primarily cater to the execution of basic tasks with specific objectives, not covering the full functionality that these platforms provide. To fully leverage the extensive capabilities of these platforms, it is imperative to acquire a more comprehensive and specialized knowledge base. Such expertise is typically obtained through dedicated courses or training programs, which may or may not involve financial commitments. The depth of understanding and proficiency achieved through such education directly correlates with the ultimate quality of the resulting product.

While 3D printed models offer notable advantages, establishing an in-house production of such tools still comes with certain limitations. In addition to the substantial knowledge and time required to proficiently use the various software programs involved, there are also associated costs inherent to the printing processes. The initial acquisition of a 3D printer, along with the requisite post-processing equipment and printing materials, entails a significant financial commitment. This investment is only sustainable if reimbursed through the inclusion of the production value of the models in the medical-veterinary services provided, which makes the 3D printing modality unattractive, or even inconceivable, for some clients, at least for now. Although the cost of printing may seem expensive, it’s crucial to consider the diverse range of available materials. Depending on the intended purpose of the printed model, there are more affordable alternatives that can be explored, such as standard resin. The key lies in selecting materials that align with the specific requirements, ultimately optimizing both cost and functionality. Nonetheless, it is expected that as technology continues to advance, the cost of production will steadily decrease and therefore, 3D printing will become more economically accessible.

When dealing with SLA printing technology, certain difficulties may arise when attempting to replicate intricate geometric conditions, particularly with delicate, thin, or hollow structures. Overcoming these challenges often requires geometry repairing or modification to ensure the accuracy and success of the printing process. Furthermore, contrary to common perception, 3D printers do not produce ready-to-use models. The post-processing, especially with SLA printers, requires a significant time investment, and also some patience, particularly when it comes to removing support structures. This becomes more pronounced when dealing with complex anatomical structures, as accessing internal support structures can present heightened challenges. Support structures can be removed at any time after printing, but the authors advise removal soon after printing, when the supports have not yet fully cured. Inadequate removal techniques can compromise the geometric accuracy of the models.

The creation of 3D models in this study varied in preparation time, depending on the species, breed, and size of the animal, averaging approximately 3 h and 30 min. The subsequent 3D printing process ranged between 8 and 20 h. These procedures, though time-consuming, seamlessly integrated into the routine pre-surgical preparations and did not disrupt patient care workflows significantly. However, it’s worth noting that in very young animals, an extended duration between model design and surgery could potentially lead to reduced accuracy in the produced model due to the animal’s growth. In the present case series, this issue was not observed. Considering this concern, in-house production may prove advantageous as it accelerates the timeline from conceptualization to 3D printing, thereby helping to mitigate such problem.

Finally, it is important to address the safe handling and proper disposal of consumables. In their liquid form, these substances raise concerns regarding user toxicity due to skin irritant properties, potential allergenicity, and the risk of respiratory irritation. Volatile organic compounds in these consumables can be irritating to the respiratory tract, potentially leading to headaches (24, 25). Therefore, it is recommended to manipulate such materials with gloves, use protective glasses, ensure room ventilation, and consider using a respiratory filter when dealing with high amounts of resin in its liquid form. Since ingestion of the liquid form is also toxic, the environmental risk is greater if the disposals are not properly discarded. Liquid 3D printing resins are considered toxic waste as they are known pollutants and cannot be discarded into the environment. Biobased resins are less harmful to the environment even in their liquid form compared to classic and older SLA resins. They are being used more frequently as new, more environmentally friendly alternatives (26). After polymerization, toxicity is low or negligible, and there are numerous biocompatible resins with FDA approval for use in medical devices for long contact with the body (27).

## Data availability statement

The original contributions presented in the study are included in the article/supplementary material, further inquiries can be directed to the corresponding author.

## Ethics statement

The work described in this article involved the use of client-owned animals, adhering to internationally recognized standards of veterinary clinical care for individual patients. While ethical approval was not deemed mandatory for this study, in accordance with local legislation and institutional requirements, it was nevertheless obtained from a committee. Written informed consent was acquired from the owners for their animals’ participation in this study. However, no animals or individuals are identifiable in this publication, and additional informed consent for publication was not considered necessary.

## Author contributions

MRG: Conceptualization, Data curation, Formal analysis, Investigation, Methodology, Writing – original draft, Writing – review & editing. LAM: Conceptualization, Data curation, Formal analysis, Funding acquisition, Investigation, Methodology, Resources, Supervision, Writing – review & editing.

## Footnotes

^1^ http://www.slicer.org
